# Purine-Induced IFN-γ Promotes Uric Acid Production by Upregulating Xanthine Oxidoreductase Expression

**DOI:** 10.3389/fimmu.2022.773001

**Published:** 2022-01-27

**Authors:** Huanhuan Wang, Lingzhu Xie, Xuhong Song, Jing Wang, Xinyan Li, Zhike Lin, Ting Su, Bin Liang, Dongyang Huang

**Affiliations:** ^1^ Department of Cell Biology and Genetics, Key Laboratory of Molecular Biology in High Cancer Incidence Coastal Chaoshan Area of Guangdong Higher Education Institutes, Shantou University Medical College, Shantou, China; ^2^ Department of Clinical Laboratory Medicine, Mianyang Central Hospital, Mianyang, China; ^3^ Research Center of Translational Medicine, Second Affiliated Hospital of Shantou University Medical College, Shantou, China

**Keywords:** purine, hyperuricemia, xanthine oxidoreductase, IFN-γ, STAT1, IRF1

## Abstract

**Objective:**

Limiting purine intake, inhibiting xanthine oxidoreductase (XOR) and inhibiting urate reabsorption in proximal tubule by uricosuric drugs, to reduce serum uric acid (UA) levels, are recognized treatments for gout. However, the mechanism of increased how XOR expression and activity in hyperuricemia and gout remains unclear. This study aims to explore whether exogenous purines are responsible for increased XOR expression and activity.

**Methods:**

HepG2 and Bel-7402 human hepatoma cells were stimulated with exogenous purine, or were exposed to conditioned growth medium of purine-stimulated Jurkat cells, followed by measurement of XOR expression and UA production to determine the effect of lymphocyte-secreted cytokines on XOR expression in hepatocytes. The expression of STAT1, IRF1 and CBP and their binding on the *XDH* promoter were detected by western blotting and ChIP-qPCR. The level of DNA methylation was determined by bisulfite sequencing PCR. Blood samples from 117 hyperuricemia patients and 119 healthy individuals were collected to analyze the correlation between purine, UA and IFN-γ concentrations.

**Results:**

Excess of purine was metabolized to UA in hepatocyte metabolism by XOR that was induced by IFN-γ secreted in the conditioned growth medium of Jurkat cells in response to exogenous purine, but it did not directly induce XOR expression. IFN-γ upregulated XOR expression due to the enhanced binding of STAT1 to IRF1 to further recruit CBP to the *XDH* promoter. Clinical data showed positive correlation of serum IFN‐γ with both purine and UA, and associated risk of hyperuricemia.

**Conclusion:**

Purine not only acts as a metabolic substrate of XOR for UA production, but it induces inflammation through IFN-γ secretion that stimulates UA production through elevation of XOR expression.

## Background

Hyperuricemia is a metabolic disease caused by abnormal purine metabolism and due to an excessively increased serum urate concentration that can occur as a result of overproduction of uric acid (UA) and/or over reabsorption of urate, possibly driven by intracellular nicotinate (1). Xanthine oxidoreductase (XOR), the rate-limiting enzyme for UA production, is widely expressed in the gut, mammary epithelial cells, kidney and myocardium, and especially in the liver, that catalyzes the conversion of hypoxanthine (hXan) to xanthine (Xan) and the latter to UA (2–6). In mammals XOR is present in two forms, initially as xanthine dehydrogenase (XDH, EC 1.17.1.4), which can be readily converted to xanthine oxidase (XO, EC 1.17.3.2) either irreversibly by proteolysis or reversibly by modification of Cys residues (3, 7). Although the reason for the low expression and activity of XOR in humans is not entirely clear (8, 9), the expression and activity of XOR are significantly increased in hyperuricemia (10–12) due to unknown regulatory mechanisms.

Elevated serum UA levels caused by a high purine diet is the most common cause of acute gout. It has become almost a medical consensus that gout patients should avoid alcohol and sweet beverages, as well as high purine foods (13–15). However, whether enhanced catalytic conversion of excess purine into UA through upregulation of XOR in hyperuricemia has not been reported. In addition, high purine and purine derivative intake not only increases serum UA, but also associated with physiological inflammatory/anti-inflammatory activity (16–18). Long-term, high-level caffeine (whose main metabolite is paraxanthine) consumers often have elevated levels of IFN-γ and TNF-α (19). The upregulation of XOR expression, by cytokines IL-1, IL-6 and IFN-γ, has been reported in lung, renal, and mammary epithelial cells (20–23), but their effect on XOR expression and UA production in hepatocytes remained unknown. Therefore, we hypothesized that purine may directly or indirectly *via* inflammatory cytokines regulate XOR expression.

IFN-γ is produced mainly by Natural killer lymphocytes (NK cells) and activated T cells (24, 25). IFN-γ binds to the interferon-γ receptor (IFNGR) on the target cell membrane to activate the classical JAK/STAT pathway. Interferon regulatory factors (IRFs) are a class of multifunctional transcription factors that bind to response elements on promoter of target genes and regulate target gene expression (26–28). They also interact with other transcription factors such as signal transducers and activators of transcription (STATs) (26, 27, 29). Moreover, the protein complex containing IRF1 further recruits histone acetyl transferases (30, 31). Though macrophages and neutrophils are considered to be the principal inflammatory cells involved in gout, some T cell subsets are also closely linked to gouty arthritis (32). In potassium oxonate-treated hyperuricemic mice, the level of inflammatory cytokines IL-1β and IFN-γ secreted by Th1 cells in the spleen is higher and the level of IL-4 and IL-10 secreted by Th2 cells is lower compared to their normal counterpart (33).

Elevation of XOR expression and activity plays an important role in hyperuricemia and gout, but the underlying mechanisms of how purine-rich diet induces inflammation and hyperuricemia remain largely unclear. In this report, we demonstrate that excessive purine generates UA without directly affecting XOR expression and activity in hepatic cells, instead indirectly elevates XOR expression induced by IFN-γ that is secreted form lymphocyte (Jurkat cells) in response to excessive purines. IFN-γ activates STAT1 and cooperates with IRF1 to recruit CBP to the *XDH* promoter in hepatocytes to upregulate XOR expression.

## Materials and Methods

### Cell Culture

The human hepatocellular carcinoma (HCC) cell lines HepG2 and Bel-7402 (Shanghai, China) were cultured in Dulbecco’s modified Eagle’s medium supplemented with 10% fetal bovine serum (Gibco, USA). The Jurkat T lymphocytic leukemia cell line (Shanghai, China) was cultured in RPMI1640 supplemented with 10% fetal bovine serum. All cell lines were cultured in a humidified atmosphere at 37°C with 5% CO_2_. Xanthine (Xan) (#69-89-6, Sangon Bio, China), hypoxanthine (hXan) (#68-94-0, Sangon Bio, China), IL-1 (#200-01B, PeproTech, China) IL-6 (#200-06, PeproTech, China), IFN-γ (#HY-P7025, MedChemExpress, China) and cycloheximide (CHX) (#HY-12320, MedChemExpress, China) were used to treat cells as indicated.

### Uric Acid Assay

Intracellular UA concentrations were measured with a Uric Acid Assay Kit (#MAK077, Sigma) as follows: HCC cells were plated at 1×10^5^ cells per well in a 6-well plate, and UA was extracted with RIPA (radioimmunoprecipitation assay buffer) at 70% to 80% confluences. Then, 50 μl cell lysate and 50 μl Master Reaction Mix were added to each well in a 96-well plate and incubated at 37°C for 30 min away from light. The absorbance at 570 nm was determined. Intracellular UA concentrations was normalized to protein concentration. Serum UA concentrations were detected with a Roche Cobas c701 biochemical analyzer according to the instructions of the Roche Uric Acid Assay Kit.

### XOR Activity Assay

XOR and XO activity in the HCC cells were determined by a Xanthine Oxidase Activity Assay Kit (#MAK078, Sigma), which results in a fluorometric (λex = 535/λem = 587 nm) product. XOR and XO activities in the HCC cells were determined by following the increase of fluorescence by using xanthine as the substrate, in the presence of NAD^+^ (for XOR) or absence of NAD^+^ (for XO), using only molecular oxygen as an electron acceptor, for the fixed time interval. The XDH activity was calculated by subtracting from XOR the XO activity (34, 35). One unit (U) of XOR was defined as the amount of enzyme that catalyzes the oxidation of xanthine to yield 1.0 μM of UA and hydrogen peroxide per minute at 25°C. XOR activity was normalized by protein concentration.

### ELISA

IFN-γ was measured with a Human IFN-γ ELISA kit (#CHE0017, 4A Bio, Chain) according to the manufacturer’s instructions. Absorbance was measured at 450 nm. The kit was designed for detection of IFN-γ in human serum and cell culture samples.

### RNA Extraction, RT-PCR and qPCR

Total RNA was isolated using Trizol (Takara, No.9109) according to the manufacturer’s instructions. Reverse transcription was performed using a PrimeScript™ RT reagent kit with gDNA Eraser (#RR047A, Takara). Quantitative real-time PCR was performed using AceQ qPCR SYBR Green Master Mix (Low ROX Premixed) (#Q131–02, Vazyme) and a QuantStudio 12 K Flex Real-Time PCR System (Thermo Fisher, USA) according to the manufacturer’s protocols. Intron-spanning primers we designed are as follows: XOR forward: 5’-GCTCTGAAAATCCCCACCTC-3’; reverse: 5’- GGAGCCACTGGGATTCTTCTT-3’; IFN-γ forward: 5’-GAGTGTGGAGACCATCAAGGAAG-3’; reverse: 5’-TGCTTTGCGTTGGACATTCAAGTC-3’; IL-1 forward: 5’-CCACAGACCTTCCAGGAGAATG-3’; reverse: 5’-GTGCAGTTCAGTGATCGTACAGG-3’; IL-6 forward: 5’-AGACAGCCACTCACCTCTTCAG-3’; reverse: 5’-TTCTGCCAGTGCCTCTTTGCTG-3’; GAPDH forward: 5’-GTTTTTCTAGACGGCAGGTCA-3’; reverse: 5’-AACATCATCCCTGCCTCTACT-3’.

### RNA Interference (RNAi) Assay

Cells were transfected with Small interfering RNA (siRNA) using Lipofectamine RNAiMAX (#13,778,100, Invitrogen, USA) according to the manufacturer’s instructions. The cells were harvested for further analysis 48 h after transfection. All siRNAs were designed by GenePharma (Shanghai, China). The sequences of the siRNAs used in this study were as follows: siSTAT1: 5’-CUCAUUCCGUGGACGAGGU-3’, siIRF1: 5’- CAGAUUAAUUCCAACCAAA-3’; siCBP: 5’-CCAUUUCUCCUUCCCGAAU-3’; siNC: 5’-UUCUCCGAACGUGUCACGU-3’.

### Western Blotting

Cell lysates were made using RIPA buffer. Equal amounts of protein (40 μg) were electrophoresed on SDS-PAGE and transferred to a PVDF membrane (Millipore, USA). Membranes were incubated overnight at 4°C with primary antibody. Antibodies used for western blotting were as follows: anti-XOR (#sc-398548, Santa Cruz, CA), anti-IFNGR1 (#ab134070, Abcam, Cambridge, MA), anti-CBP (#sc-7300 X, Santa Cruz, CA), anti-STAT1 (#14994, Cell Signaling Technology (CST), Danvers, MA), anti-pSTAT1 (#9167, CST, Danvers, MA), anti-STAT3 (#9139, CST, Danvers, MA), anti-pSTAT3 (#9145, CST, Danvers, MA), anti-IRF1 (#8478, CST, Danvers, MA), and anti-β-actin (#sc-130656, Santa Cruz, CA). HRP-conjugated secondary antibody was incubated for 2 h at room temperature. Protein bands were analyzed using a Bio-Rad Gel Doc system with Image Lab software.

### Chromatin Immunoprecipitation (ChIP)

ChIP-qPCR was used to detect histone modification and transcription factors (TFs) binding within the promoter region of *XDH*. As we described before (36), the ChIP assay was performed using a Magna ChIP™ G Chromatin Immunoprecipitation Kit (#17-611, Millipore-Sigma, Burlington, MA) according to the manufacturer’s protocol. ChIP-grade antibodies were as follows: anti-H3K27ac (#720096, Thermo Fisher Scientific, Bedford, MA), anti-CBP (#PA1–847, Thermo Fisher, Bedford, MA), anti-STAT1 (#14994, CST, Danvers, MA), and anti-IRF1 (#8478, CST, Danvers, MA). Immunoprecipitated DNA was detected by qPCR and normalized to input DNA.

### Bisulfite Sequencing PCR (BSP)

DNA methylation was examined by bisulfite conversion of extracted genomic DNA with a bisulfite conversion kit (#DP215, TIANGEN, China). The *IFN-γ* promoter was amplified by PCR using a methylation-specific PCR kit (#EM101, TIANGEN, China). Primer sequences were designed using Methyl Primer Express v1.0 (Applied Biosystems, USA), and Sanger sequencing was performed on the PCR products. Five individual PCR products were sequenced for each sample. Primers we designed are as follows: IFN-γ-BSP forward: 5’- TTTAGTATTTTGGGAGGTTAAGG-3’; reverse: 5’-ATCACCCAAACTAAAATACAATAAC-3’.

### Patients and Serum

For this study, we collected 236 cases of blood samples from 117 hyperuricemic patients and 119 healthy individuals in the Mianyang Central Hospital, Sichuan Province, China during 2018-2019. Males with UA > 420 μmol/L and females with UA > 360 μmol/L and no history of gout were selected for the study. All blood samples were centrifuged at 2,000 g for 10 min, and the serum samples were taken for storage at -80°C. This study was approved by the Medical Ethics Committee of Shantou University Medical College (SUMC-2020-13).

### LC/MS

One to two volumes of acetonitrile were added to each serum sample, followed by vigorous shaking and then centrifugation at 10,000g for 10 min to precipitate proteins. Then the supernatant was filtered to prepare for loading. Xanthine (Xan), hypoxanthine (hXan), acetonitrile and formic acid were purchased from Thermo Fisher.

For measurement of Xan and hXan in human serum samples, a Shimadzu Prominence UFLC + LCMS-2020 was used. Separation was performed on a Phenomenex Luna C18 column (4.6 × 150 mm, 5 μm) with the column oven temperature set at 40℃. Then, 0.1% formic acid aqueous solution (A) and acetonitrile (B) were used as the mobile phases for gradient elution. Spectra of the full mass scans indicated that Xan and hXan derivatives exhibited precursor ions [M]+ at 153 m/z for Xan and 173 for hXan.

### Statistical Analysis

Statistical analyses were conducted using SPSS 19.0 (IBM Corporation, USA) and GraphPad Prism 7.0 (GraphPad, CA). All data shown were determined from three independent experiments unless otherwise stated, and presented as the mean ± S.D., with *p*-values presented as *p < 0.05, **p < 0.01, ***p < 0.001. In addition, Spearman’s correlation and a multiple linear regression model were used to evaluate the correlation and dose-response relationship between purine, IFN-γ and UA after adjusting for covariates, including age, gender, aspartate amino transferase (AST), alanine amino transferase (ALT), total cholesterol (TC), triglyceride (TG), and random blood glucose (Glu).

## Results

### Exogenous Purine Elevates UA Production in Hepatocytes Without Affecting XOR Expression

**Figure 1:**
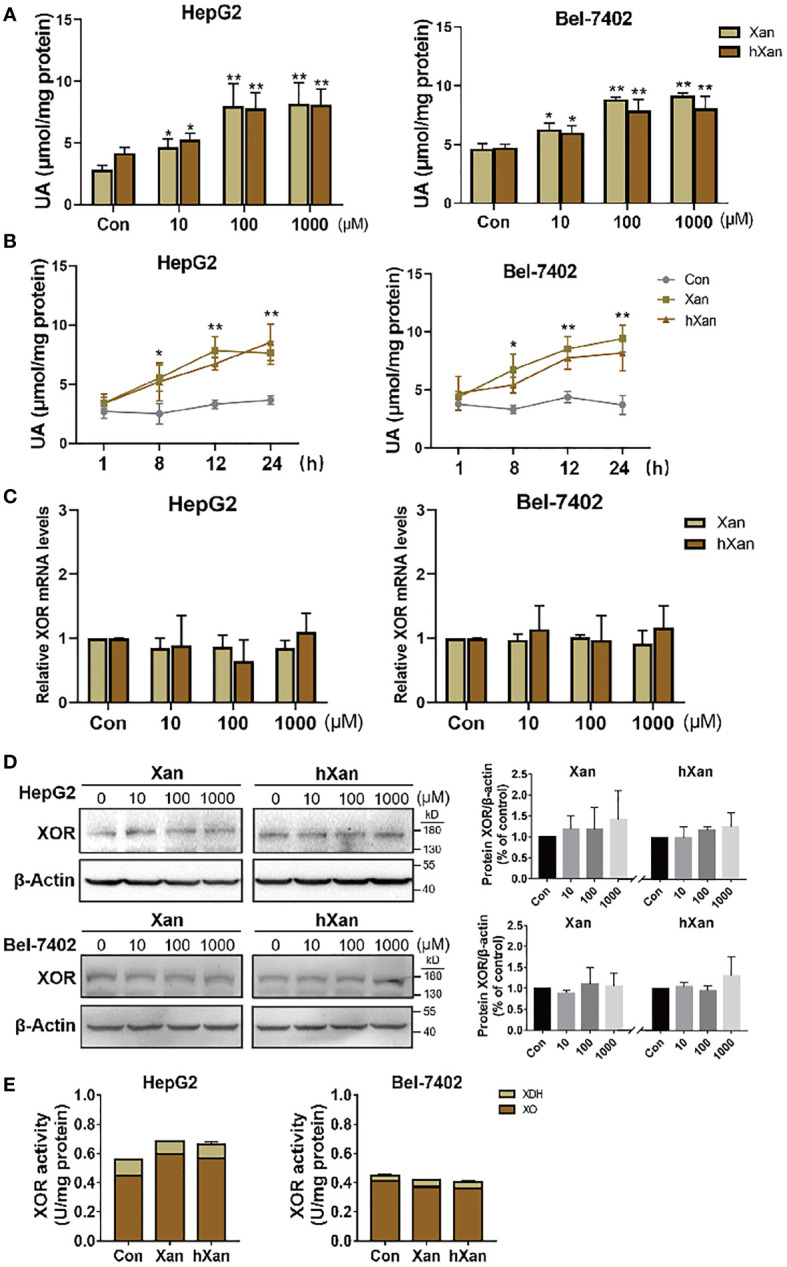
Exogenous purines elevate UA production in hepatic cell lines without affecting XOR expression. **(A)** HepG2 and Bel-7402 cells were maintained in purine for 24 h at different concentrations, or **(B)** 100 µM purine for varying intervals of time, and then UA levels were determined by a coupled enzyme assay. **(C)** Expression of XOR mRNA levels, **(D)** protein levels and **(E)** XOR activity were detected in HepG2 and Bel-7402 cells after maintenance in 100 μM purine for 24 h. Significance for all data was determined by the independent samples *t*-test. Data are shown as mean ± S.D., *p < 0.05, **p < 0.01.

HepG2 and Bel-7402 cells were plated at 1×10^5^ cells per well in a 6-well plate and allowed to attach overnight. Then, cells were treated with varying concentrations of xanthine (Xan) or hypoxanthine (hXan) for 24 h and then intracellular UA concentrations were measured. We found that intracellular UA concentrations were increased in a concentration-dependent manner and then plateaued at 100 μM Xan and hXan (**Figure 1A**). When cells were treated with 100 μM purine for varying intervals of time, intracellular UA concentrations were increased in a time-dependent manner (**Figure 1B**). These results indicate that exogenous purine elevates intracellular UA production in cultured hepatocytes and that UA production increased with increasing concentrations of substrate and plateaued at substrate concentration of 100 μM, which could be the result of saturation of substrate binding site of XOR leading to a steady state. To check the effect of purine on XOR expression, we measured XOR expression and the activity after treatment with excess purine (100 μM, 24 h). The results of qPCR and western blotting analysis of HepG2 and 7402 cells showed that treatment with 100 μM purine for 24 h did not affect XOR mRNA and protein expression (**Figures 1C, D**). Analysis of enzyme activity revealed that XOR activity was also remained unchanged after treatment with purine (**Figure 1E**). XO activity is higher than XDH activity, possibly due to the readily conversion of XDH to XO under the action of the intracellular environment (7). The results indicate that exogenous purine elevates UA production in hepatocytes without affecting XOR expression.

### Exogenous Purine Increases the Expression of IFN-γ in Lymphoblasts (Jurkat Cells)

**Figure 2:**
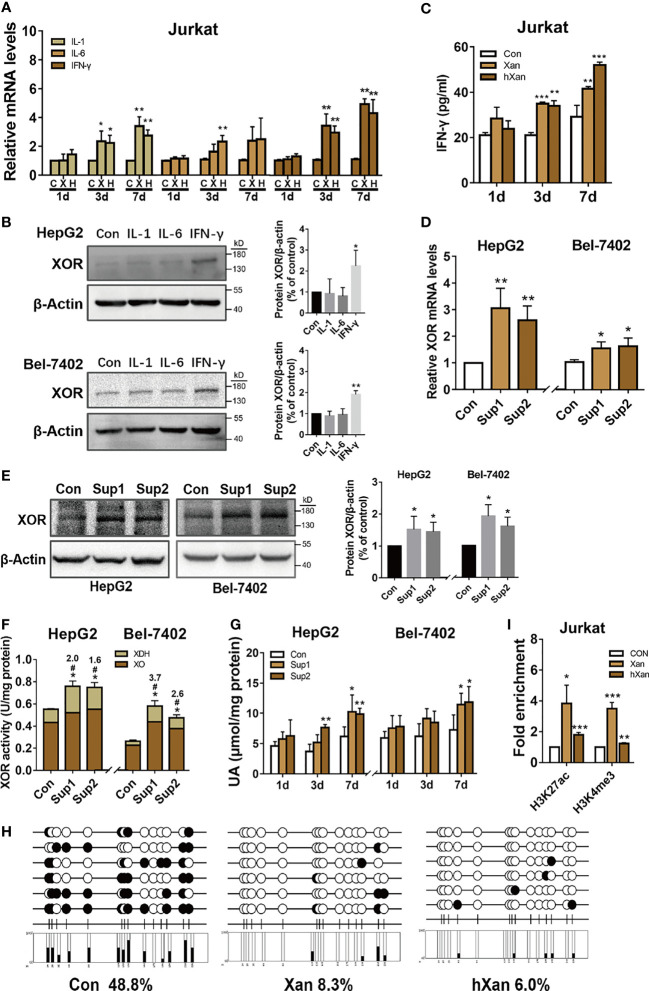
IFN-γ secreted from lymphoblast (Jurkat cells) upon induction by exogenous purine upregulates XOR expression in hepatic cell lines. **(A)** The expression levels of mRNAs for IL-1, IL-6 or IFN-γ in Jurkat were determined by qPCR after treatment with 100 μM purine for 1 to 7 days. **(B)** The expression levels of XOR protein in HepG2 and Bel-7402 cells were determined by western blotting after treatment with IL-1, IL-6 or IFN-γ for 24 h. **(C)** Levels of IFN-γ in the culture medium of Jurkat cells were determined by ELISA after treatment with 100 μM Xan and hXan for 1 to 7 days. **(D, E)** HepG2 and Bel-7402 cells were cultured for 24 h in conditioned growth medium after 7 days of growth of Jurkat cells in presence of 100 μM purines. The expression levels of XOR mRNA and protein in hepatic cells were determined by qPCR and western blotting respectively. **(F, G)** The XOR activity and intracellular UA levels were determined by enzyme coupling assay. **(H)** The DNA methylation level of CpG islands (black circle: methylated, white circle: unmethylated) in the *IFN-γ* promoter were determined by BSP, and **(I)** the enrichment levels of H3K27ac and H3K4me3 in the *IFN-γ* promoter were assessed by ChIP-qPCR after treatment of Jurkat with Xan and hXan for 7 days. Significance for all data determined by the independent samples *t*-test. Data are shown as mean ± S.D., *p < 0.05, **p < 0.01, ***p < 0.001. **(F)** *p < 0.05 compared with Con XOR, ^#^ *p* < 0.05 compared with Con XDH.

Diets high in fat, purine, and sugar can increase the risk inflammation (16–18) and gout (37), the disease preceded by hyperuricemia. Inflammatory cytokines like IL-1, IL-6 and IFN-γ have been reported in lung, renal, and mammary epithelial cells to upregulate of XOR expression but their effect on hepatocytes is unknown (20–23). Therefore, we hypothesized that inflammation induced by high-purine intake may be involved in the pathogenesis of hyperuricemia. To explore this possibility, Jurkat cells were plated at 5×10^5^ cells per well in a 6-well plate and allowed to grow overnight. Then, cells were treated with 100 μM purine for 0 to 7 days. The purine-containing medium was replaced every 2 days. The results of qPCR analysis of cytokine expression showed that the expression levels of mRNAs for IL-1, IL-6 and IFN-γ were increased in purine-treated Jurkat cells to varying degrees, and in a time-dependent manner (**Figure 2A**). Next, HepG2 and Bel-7402 cells were treated with IL-1, IL-6 or IFN-γ to verify the effect of cytokines on XOR expression in hepatocytes. Western blotting analysis showed that XOR expression in HepG2 and Bel-7402 cells was significantly increased (2.2-fold and 1.9-fold, respectively) only with IFN-γ treatment, but not with IL-1 or IL-6 (**Figure 2B**). In addition, ELISA analysis of Jurkat cell culture medium showed that IFN-γ levels were indeed elevated after purine treatment for 3 to 7 days (**Figure 2C**).

To test the effect of IFN-γ, secreted in the conditioned growth medium by purine-induced lymphoblast (Jurkat cells) for 7 days, on HepG2 and Bel-7402 cells, we assessed the XOR mRNA and protein expression by qPCR and western blotting analyses respectively after 24 h of culture. The results show increased expression of both XOR mRNA (**Figure 2D**) and protein (1.5 to 2.2-fold) (**Figure 2E**) in HepG2 and Bel-7402 cells. In addition, we found an increase in total XOR activity, with a 1.7 to 3.7-fold increase in XDH activity, while XO activity remained unchanged (**Figure 2F**). IFN-γ-induced XDH can also be partially converted to XO, but the activity of XDH increases more than XO, which may be related to the conversion rate. These results suggest that the increase in XOR activity and expression may be dominated by the XDH form that had not yet been converted to the XO form. Moreover, intracellular content of UA in HepG2 and 7402 cells also showed gradual increase over the 1 to 7 days following culture in the conditioned growth medium (**Figure 2G**).

To explore the mechanism of how exogenous purine-induced IFN-γ production in lymphocytes, we treated Jurkat cells with 100 μM of Xan and hXan for 7 days, and then bisulfite sequencing PCR (BSP) was used to detect the DNA methylation level of the CpG island in the *IFN-γ* promoter. In addition, ChIP-qPCR was used to detect histone acetylation and methylation levels in the *IFN-γ* promoter. The results showed that the DNA methylation level in the CpG island of *IFN-γ* promoter was reduced (**Figure 2H**), and the enrichment of H3K27ac and H3K4me3 were increased in the Xan and hXan groups (**Figure 2I**). These results show that DNA methylation and histone modifications of the *IFN-γ* promoter were likely to be associated with purine-induction of IFN-γ expression. Overall, the above results suggest that purine-induced IFN-γ production by lymphocytes upregulates XOR expression in hepatocytes leading to elevated UA production.

### IFN-γ Regulates XOR Expression *via* JAK/STAT1 Signaling Pathway

**Figure 3:**
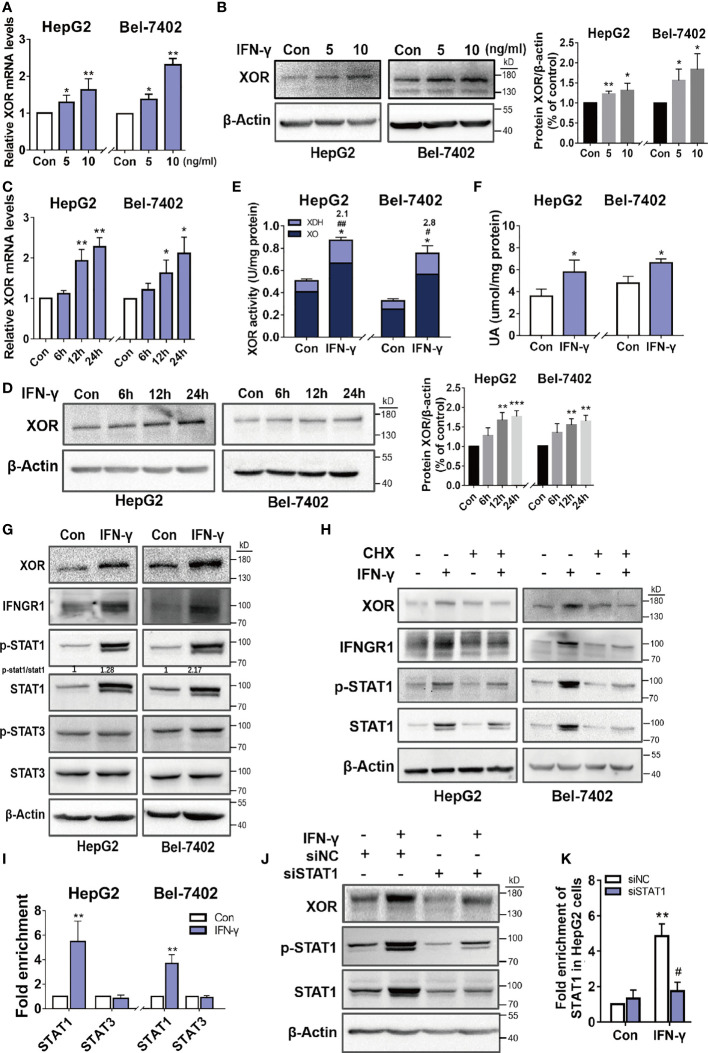
IFN-γ upregulates XOR expression by stimulating expression and activation of the STAT1. **(A-D)** HepG2 and Bel-7402 cells were incubated with varying concentrations of IFN-γ for varying time intervals and then XOR mRNA and protein expression were measured by qPCR and western blotting respectively. **(E, F)** HepG2 and Bel-7402 cells were treated with IFN-γ (10 ng/ml) for 24 h and then XOR activity and intracellular UA content were measured by enzyme coupling assay. **(G)** The protein expression levels of XOR, IFNGR1, STAT1 and STAT3 were examined by western blotting and **(I)** the enrichment of STAT1 and STAT3 were assessed by ChIP-qPCR. **(H)** The effect of IFN-γ (10 ng/ml) on the expression of XOR, IFNGR1 and STAT1 proteins were examined after pre-treatment with cycloheximide (CHX) (500 nM) for 24h. **(J, K)** After transfection with siRNA for 48h, HepG2 cells were treated with IFN-γ (10 ng/ml) and then the expression of XOR and STAT1 proteins were determined by western blotting. **(K)** The enrichment of STAT1 at the *XDH* promoter were assessed by ChIP-qPCR. Significance for all data determined by the independent samples *t*-test. Data are shown as mean ± S.D., *p < 0.05, **p < 0.01. **(E)** *p < 0.05 compared with control XOR, ^#^ *p* < 0.05, ^##^ *p* < 0.01 compared with control XDH. **(K)** *p < 0.05 compared with control siNC (negative control siRNA); ^#^ *p* < 0.05 compared with IFN-γ siNC.

To explore the intracellular mechanism of how IFN-γ induces XOR expression in hepatic cells, we examined the expression levels of XOR mRNA by qPCR and XOR protein by Western blotting in HepG2 and Bel-7402 cells treated with recombinant human IFN-γ. The expression of XOR mRNA and protein were found to increase in a time- and dose-dependent manner (**Figures 3A–D**). We also found increase in XOR activity and intracellular UA content (**Figures 3E, F**). Analysis of enzyme activity showed increase of only XDH activity (2.1- to 2.8-fold) which could be due to the increase in XOR protein expression level (1.2- to 2.2-fold) (**Figures 3B, D**), while the XO activity remained unchanged. These results indicate that IFN-γ enhances UA production by upregulating XOR expression in HepG2 and Bel-7402 cells.

The JAK/STAT (Janus kinase/signal transducer and activator of transcription) pathway is a classical signaling pathway activated by IFN-γ. Western blotting analysis of HepG2 and Bel-7402 cells after IFN-γ treatment revealed increased expression of IFNGR1, p-STAT1 and STAT1, and an increased ratio of p-STAT1/STAT1, whereas the protein level of p-STAT3 and STAT3 were unchanged (**Figure 3G**). However, after pre-treatment with cycloheximide (CHX), a protein synthesis inhibitor, the IFN-γ-induced upregulation of XOR, IFNGR1, p-STAT1 and STAT1 expression were abolished (**Figure 3H**).

To determine whether STAT1 is involved in the upregulation of XOR expression as a transcriptional activator, ChIP-qPCR was used to examine the binding of STAT1 to the *XDH* promoter. As shown in **Figure 3I**, the enrichment of STAT1 were increased by IFN-γ treatment compared to the control, while STAT3 enrichment were unchanged. Furthermore, IFN-γ-induced XOR expression was reduced after STAT1 knockdown by siRNA that corresponded to the reduction of STAT1 population at the *XDH* promoter (**Figures 3J, K**). These results suggest that IFN-γ upregulates XOR expression by stimulating expression and activation of the STAT1 rather than STAT3, and that activated STAT1 bind to the *XDH* promoter to stimulate XOR expression.

### STAT1-IRF1 Recruits CBP to Co-Regulate *XDH* Transcription

**Figure 4:**
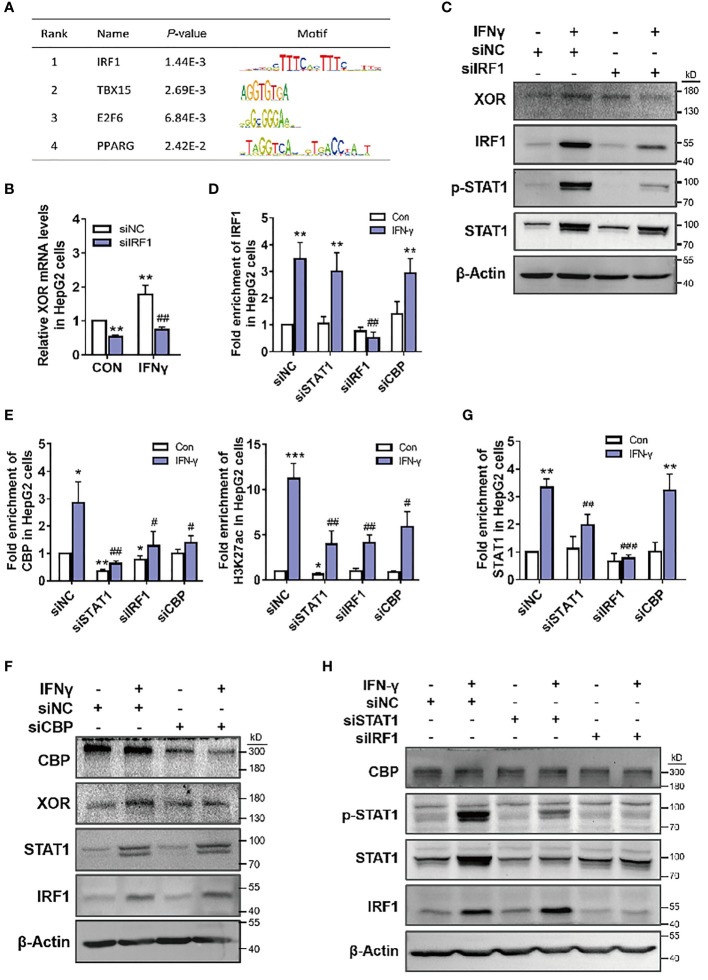
CBP is recruited by STAT1-IRF1 to upregulate XOR expression in HepG2 cells. **(A)** Analysis of the *XDH* promoter sequence using transcription factor affinity prediction (TRAP) web tools (http://trap.molgen.mpg.de/cgi-bin/trap_multi_seq_form.cgi). **(B, C)** XOR mRNA and protein expression, after treatment with siNC or siIRF1 for 48 h; **(D)** IRF1, **(E)** CBP and H3K27ac, **(G)** STAT1 enrichment after treatment with siNC, siIRF1, siSTAT1 for 48 h or with siCBP for 24 h; **(F)** CBP, XOR, STAT1 and IRF1 protein expression after treatment with siNC or siCBP for 24 h; **(H)** CBP, p-STAT1, STAT1, and IRF1 protein expression after treatment with siNC, siSTAT1 or siIRF1 for 48 h, in HepG2 cells treated with or without IFN-γ (10 nM). Significance for all data was determined by the independent samples *t*-test. Data are shown as mean ± S.D., *p < 0.05, **p < 0.01 compared with Control; ^#^ *p* < 0.05, ^##^ *p* < 0.01, ^###^ *p* < 0.001 compared with IFN-γ.

To further explore the regulation of XOR expression, we analyzed the sequence of *XDH* promoter using transcription factor affinity prediction (TRAP) web tools. We identified four plausible transcription factors (TFs) with statistically significant *p*-values (**Figure 4A**). To verify whether IRF1 is involved in IFN-γ-induced *XDH* transcription, we stimulated hepatocytes with recombinant human IFN-γ and determined expression of IRF1 and its binding at the *XDH* promoter. The results showed increase in the expression of IRF1 mRNA and protein after treatment with IFN-γ and decrease in the IFN-γ-induced XOR expression after IRF1 knockdown (**Figures 4B, C**). Furthermore, the enrichment of IRF1 at the *XDH* promoter was increased after IFN-γ stimulation, whereas the enrichment was reduced after IRF1 knockdown (**Figure 4D**). These results indicate that IRF1 is indeed involved in IFN-γ-induced XOR expression through regulating *XDH* transcription by binding to the *XDH* promoter.

In the above experiments, we also found that with knockdown of STAT1, enrichment of CBP histone acetyltransferase at the *XDH* promoter decreased accordingly (**Figure 4E**). To validate whether STAT1 activates *XDH* transcription by affecting CBP binding, we knocked down CBP by siRNA and measured XOR expression in HepG2 cells. The results showed that IFN-γ-induced XOR protein levels decreased after CBP knockdown (**Figure 4F**). Moreover, CBP enrichment was statistically reduced at the *XDH* promoter, and caused a decrease in H3K27ac enrichment (**Figure 4E**). These results suggest that CBP participates in the activation of *XDH* by increasing the level of H3K27ac in the *XDH* promoter. Interestingly, STAT1 knockdown decreased both CBP and H3K27ac enrichment (**Figure 4E**), whereas CBP knockdown did not affect STAT1 enrichment (**Figure 4G**). Additionally, CBP and STAT1 knockdown did not affect each other’s expression (**Figures 4F, H**). These results suggest that enrichment of STAT1 is necessary for the recruitment of CBP and increases the accessibility of chromatin at the *XDH* promoter. However, knockdown of IRF1 reduced CBP recruitment and *XDH* promoter hyperacetylation (**Figure 4E**), but this reduction was most likely due to the decrease in STAT1 protein expression and enrichment by IRF1 knockdown (**Figures 4C, H**). In addition, STAT1 knockdown did not affect the protein expression and enrichment of IRF1, and CBP and IRF1 knockdown did not affect each other’s expression (**Figures 4F, H**). These results indicate that IFN-γ activates STAT1 and *XDH* transcription in an IRF1-dependent manner, with STAT1 and IRF1 binding to the promoter and recruiting CBP to co-activate *XDH* transcription.

### Xan, hXan and UA Levels in Human Serum Are Positively Correlated With IFN-γ

**Figure 5:**
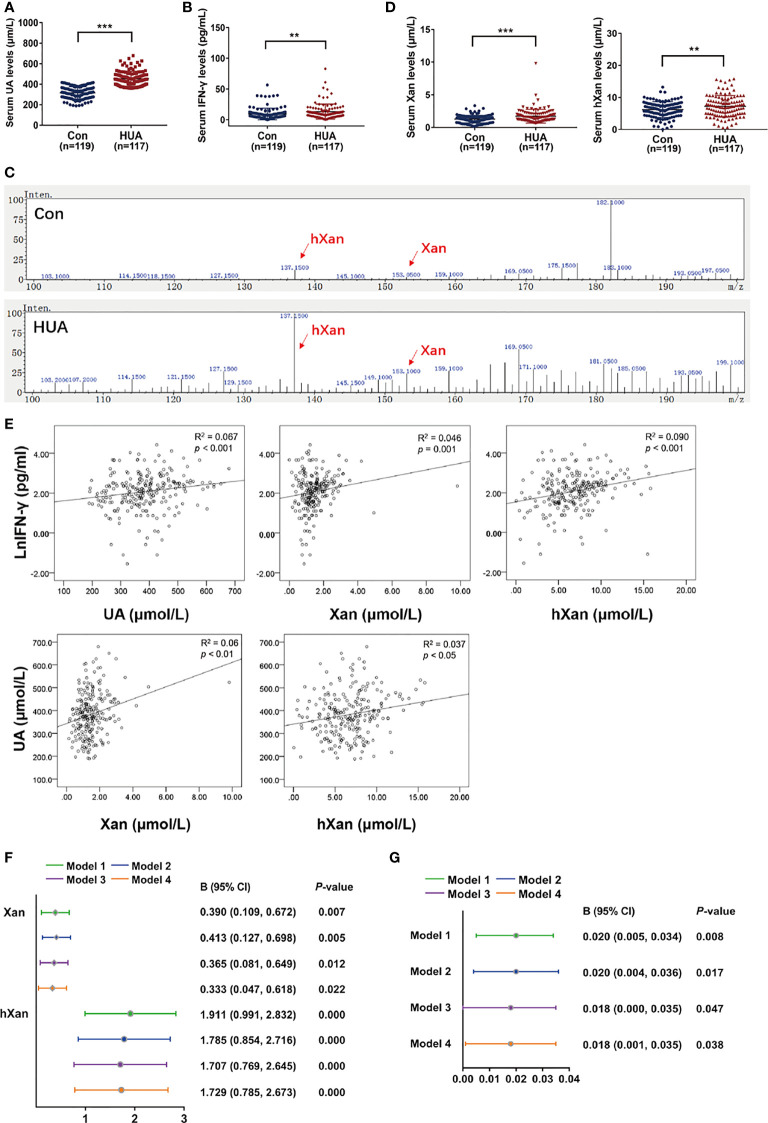
Correlation analysis of Xan, hXan, IFN-γ and UA in normal healthy individuals and hyperuricemia patients. **(A)** Serum UA levels were determined by a coupled enzyme assay. **(B)** Serum IFN-γ levels were determined by ELISA. **(C, D)** Serum Xan and hXan levels were determined by LC/MS. **(E)** Spearman’s correlation model was used to evaluate the correlation of serum Xan, hXan, UA and IFN-γ levels (natural logarithm lnIFN-γ data). **(F)** The multiple linear regression model analysis was performed between serum Xan, hXan variables and lnIFN-γ as variables. Model 1: unadjusted. Model 2: adjusted for age and gender. Model 3: adjusted for age, gender, AST, and ALT. Model 4: adjusted for age, gender, AST, ALT, TC, TG, and GLU. **(G)** Multiple linear regression model analysis was performed between IFN-γ variables and serum UA variables. Models 1-3 same as above. Model 4: adjusted for age, gender, AST, ALT, TC, and GLU. **(A–D)** Significance determined by the independent samples *t*-test. Data are shown as mean ± S.D., **p < 0.01, ***p < 0.001.

Blood samples from 236 hyperuricemia patients and healthy individuals were collected to determine serum UA, IFN-γ, and purine concentrations. The results showed that the serum UA and IFN-γ concentrations in hyperuricemic patients were significantly higher than the normal healthy individuals (control) (**Figures 5A, B**). Moreover, LC/MS measurements of purine levels showed that Xan and hXan in hyperuricemia patients were significantly higher than normal healthy individuals (**Figures 5C, D**). Spearman’s correlation analysis for purine, IFN-γ and UA showed that UA, Xan and hXan had a positive correlation with IFN-γ levels (**Figure 5E**). Multiple linear regression model analysis showed that higher levels of Xan and hXan in serum promoted the increase of IFN-γ under different adjusted models (38, 39), with hXan having a greater effect on IFN-γ production than Xan (**Figure 5F**), and that serum IFN-γ levels correlated with the increase of UA in a linear fashion (**Figure 5G**). These results show that purine has a significant effect on serum IFN-γ levels, and IFN-γ might be an important factor in the elevation of serum UA.

## Discussion

Mammalian XOR exists in large quantities predominantly in the liver. Endogenous and exogenous purine are oxidized to UA by XOR, a rate-limiting enzyme for UA production during purine metabolism. However, the mechanism of increased XOR expression and activity in patients with hyperuricemia and gout remains unknown. Gout is associated with hyperuricemia, and reducing UA levels by restriction of purine intake, XOR inhibitors and anti-inflammatory therapy are routinely used by the medical community for the treatment of gout (13–15). However, the results of the effects of exogenous purine on XOR expression in liver are not consistent between *in vivo* and *in vitro* studies. In animal experiments, the expression and activity of XOR in the liver of purine-induced hyperuricemic rats is increased (9, 10). In this study, the addition of exogenous purine to the growth medium did not directly affect the expression and activity of XOR in liver cell lines (**Figure 1**). Notably, the results of this study show an important mechanism for the dual action of purine: firstly, purines act as the substrate for XOR for conversion to UA, and secondly it stimulated UA production through enhanced production and secretion of inflammatory cytokine like IFN-γ. We show here that, purines induce XOR expression through production and secretion of IFN-γ which can explain the results of increased expression and activity of XOR in the liver of purine-induced hyperuricemic rats.

In humans, UA is the final product of purine metabolism that is largely derived from endogenous purine, with very little from exogenous sources such as foods with high purine content (37, 40, 41). Due to the absence of uricase during evolution, XOR enzyme activity in humans is 100 times lower than that in other mammals, as an adaptive mechanism (8, 9). Our results (**Figure 5D**) and previous studies show that human serum purine levels are also very low (42). When our body intake of exogenous purine is high or endogenous purine biosynthesis increases, higher level of serum purines stimulates IFN-γ production and secretion from blood lymphocytes, which in turn stimulates XOR expression in hepatocytes causing higher serum urate levels. Our clinical data also show a positive correlation between serum purine, UA and IFN-γ levels (**Figure 5E**).

A study of 91 participants showed that serum IFN-γ is not elevated in hyperuricemia and gout patients (43), which contrasts another recent study of 240 participants showing that IFN-γ is significantly elevated in the gout remission stage (44). Therefore, these seemingly contradictory results demand further investigation. Earlier studies have reported that there are patients with hyperuricemia who do not experience gout, but who show signs of systemic inflammation, suggesting that the presence of elevated serum urate may represent the presence of low-grade systemic inflammation (45, 46). Investigators have speculated that the association of UA with systemic inflammation is possibly related to a separate mediator system that both drives inflammation and increases serum UA levels (45). In this study, we demonstrate that high purine has a dual effect of not only increasing UA levels, but also driving inflammation. However, the physiological implications of this finding are unclear.

Our clinical data demonstrate that IFN-γ is associated with risk of hyperuricemia (**Figure 5G**). This data further suggest that inflammation might be a key mediator in high-purine-induced hyperuricemia and can explain the need for anti-inflammatory therapy for hyperuricemia and prevention of gout flares. Allopurinol and febuxostat are recommended as the first-line pharmacologic urate-lowering therapy for gout but are not currently prescribed for the treatment of asymptomatic hyperuricemia due to adverse effects (47). There has been a lack of effective therapeutic drugs for the treatment of asymptomatic hyperuricemia. Traditional anti-inflammatory therapy is usually used during the onset of gouty arthritis. One study has recommended anti-inflammatory prophylaxis for all gout patients when there is any clinical evidence of continuing gout disease activity (48). The results of our study suggest anti-inflammatory treatment not only as a strategy for post-onset treatment for gout, but also for asymptomatic hyperuricemia.

## Conclusion

It is well known that exogenous purine is mainly metabolized by the liver to UA. Here we provide new *in vitro* evidence that excessive purine does not directly promote XOR expression in the human liver cells, but rather acts on lymphocytes, for enhanced production and release of IFN-γ, which stimulates XOR expression in hepatocytes resulting in elevation of UA production.

## Data Availability Statement

The original contributions presented in the study are included in the article/supplementary material. Further inquiries can be directed to the corresponding author.

## Ethics Statement

This study was approved by the Medical Ethics Committee of Shantou University Medical College (SUMC-2020-13). The patients/participants provided their written informed consent to participate in this study.

## Author Contributions

HW contributed to study design and was a major contributor in writing the manuscript. LX contributed formal analysis and experimental validation. XS contributed to experimental validation and supervision. JW contributed to data collection and analysis of blood samples. XL performed the experiments and the data analyses. ZL performed the experiments and literature searches. TS contributed to project administration. BL contributed to methodology, formal analysis, and experimental supervision. DH contributed to conceptualization of the study, review and editing, and funding acquisition. All authors contributed to the article and approved the submitted version.

## Funding

This work was supported by the National Natural Science Foundation of China (31770876); Guangdong Natural Science Foundation (2020A1515010174); National and provincial Natural Science Foundation cultivation special project (210719166884337, 210719166884340); 2020 Li Ka Shing Foundation Cross-Disciplinary Research Grant (2020LKSFG09B).

## Conflict of Interest

The authors declare that the research was conducted in the absence of any commercial or financial relationships that could be construed as a potential conflict of interest.

## Publisher’s Note

All claims expressed in this article are solely those of the authors and do not necessarily represent those of their affiliated organizations, or those of the publisher, the editors and the reviewers. Any product that may be evaluated in this article, or claim that may be made by its manufacturer, is not guaranteed or endorsed by the publisher.

## Abbreviations

ALT, alanine amino transferase; AST, aspartate amino transferase; BSP, bisulfite sequencing PCR; CBP, CREB-binding protein; ChIP, chromatin immunoprecipitation; CHX, cycloheximide; GLU, glucose; HCC, human hepatocellular carcinoma; hXan, hypoxanthine; IFN-γ, interferon-gamma; IFNGR, interferon- gamma receptor; IRF1, interferon regulatory factor 1; STAT1, signal transducer and activator of transcription 1; TC, total cholesterol; TG, triglyceride; UA, uric acid; Xan, xanthine; XOR, xanthine oxidoreductase.
